# Supplementary material for: Screening for Patients with Visual Acuity Loss in Primary Health Care: A Cross Sectional Study in a Deprived Hungarian Population
Source: Healthcare (Basel). 2023 Jul 5;11(13):1941. doi: 10.3390/healthcare11131941 (PMC10341402; doi:10.3390/healthcare11131941)
Supplement: Supplementary file 1 [file healthcare-11-01941-s001.zip › healthcare-2474326-supplementary.pdf]

Table S1. Demographic composition of the studied and the target population of the screening for visual acuity loss with the yield of screening across strata

age groups	whole population		target group of the screening		positive screening results	
	males	females	males	females	Males	Females
18-19	19 (1.44%)	44 (1.73%)	14 (1.82%)	29 (2.23%)	0 (0%)	0 (0%)
20-24	62 (4.71%)	107 (4.20%)	56 (7.29%)	84 (6.45%)	0 (0%)	0 (0%)
25-29	53 (4.02%)	117 (4.59%)	42 (5.47%)	92 (7.07%)	1 (5.56%)	1 (1.72%)
30-34	72 (5.47%)	130 (5.10%)	61 (7.94%)	102 (7.83%)	1 (5.56%)	2 (3.45%)
35-39	67 (5.09%)	173 (6.79%)	54 (7.03%)	130 (9.98%)	0 (0%)	4 (6.90%)
40-44	83 (6.30%)	211 (8.28%)	69 (8.98%)	158 (12.14%)	0 (0%)	1 (1.72%)
45-49	112 (8.50%)	217 (8.52%)	77 (10.03%)	137 (10.52%)	1 (5.56%)	2 (3.45%)
50-54	141 (10.71%)	242 (9.50%)	97 (12.63%)	150 (11.52%)	1 (5.56%)	13 (22.41%)
55-59	160 (12.15%)	281 (11.03%)	84 (10.94%)	126 (9.68%)	3 (16.67%)	5 (8.62%)
60-64	158 (12.00%)	287 (11.26%)	74 (9.64%)	93 (7.14%)	5 (27.78%)	5 (8.62%)
65-69	143 (10.86%)	247 (9.69%)	60 (7.81%)	75 (5.76%)	1 (5.56%)	5 (8.62%)
70-74	112 (8.50%)	235 (9.22%)	42 (5.47%)	70 (5.38%)	1 (5.56%)	11 (18.97%)
75-79	72 (5.47%)	142 (5.57%)	23 (2.99%)	36 (2.76%)	2 (11.11%)	4 (6.90%)
80-84	43 (3.26%)	83 (3.26%)	10 (1.30%)	16 (1.23%)	1 (5.56%)	4 (6.90%)
85-89	13 (0.99%)	25 (0.98%)	4 (0.52%)	4 (0.31%)	0 (0%)	1 (1.72%)
90-94	7 (0.53%)	6 (0.24%)	1 (0.13%)	0 (0%)	1 (5.56%)	0 (0%)
95-99	0 (0%)	1 (0.04%)	0 (0%)	0 (0%)	0 (0%)	0 (0%)
total	1317 (100%)	2548 (100%)	768 (100%)	1302 (100%)	18 (100%)	58 (100%)

Table S2 Characteristics of the study population not above 65 years of age

		N (%)
Sex	Male	628 (36.3%)
	Female	1101 (63.7%)
Age	mean (±SD)	43.1 (±12.6)
Education	non-completed primary school	188 (10.9%)
	completed primary school	492 (28.5%)
	secondary school without graduation	454 (26.3%)
	secondary school with graduation	463 (26.8%)
	higher education	132 (7.6%)
Employment	Unemployed	743 (43%)
	Employed	986 (57%)
Ethnicity	non-Roma	1491 (86.2%)
	Roma	238 (13.8%)
Hypertension	Normotensive	851 (49.2%)
	untreated hypertension	232 (13.4%)
	insufficiently treated hypertension	343 (19.8%)
	properly treated hypertension	303 (17.5%)
Diabetes	Normoglycemic	1540 (89.1%)
	untreated diabetes	51 (2.9%)
	insufficiently treated diabetes	87 (5%)
	properly treated diabetes	51 (2.9%)
Visual acuity	visual acuity ≥0.5	1684 (97.4%)
	visual acuity <0.5	45 (2.6%)
**Total number of participants**		**1729**

Table S3. Association between socio-demographic and clinical characteristics with the visual impairment among adults not above 65 by multivariate linear and logistic regression models

**Influencing factors**		**adjusted linear regression coefficients***	**adjusted odds ratios***
Sex	male	0 (reference)	1 (reference)
	female	0.72 (-0.16 ; 1.59)	1.37 (0.66 ; 2.81)
Age	year	**0.12 (0.08 ; 0.16)**	**1.08 (1.04 ; 1.12)**
Education	non-completed primary school	0 (reference)	1 (reference)
	completed primary school	0.64 (-0.81 ; 2.08)	1.36 (0.57 ; 3.24)
	secondary school without graduation	-1.37 (-2.88 ; 0.14)	0.75 (0.26 ; 2.18)
	secondary school with graduation	**-1.72 (-3.23 ; -0.22)**	0.32 (0.09 ; 1.14)
	higher education	-1.61 (-3.58 ; 0.36)	1.02 (0.24 ; 4.34)
Employment	unemployed	0 (reference)	1 (reference)
	employed	**-1.15 (-1.99 ; -0.31)**	0.52 (0.26 ; 1.02)
Ethnicity	non-Roma	0 (reference)	1 (reference)
	Roma	**2.14 (0.91 ; 3.36)**	**2.3 (1.08 ; 4.93)**
Hypertension	normotensive	0 (reference)	1 (reference)
	untreated hypertension	0.38 (-0.92 ; 1.69)	0.51 (0.18 ; 1.47)
	insufficiently treated hypertension	0.31 (-0.91 ; 1.54)	0.58 (0.25 ; 1.34)
	properly treated hypertension	-0.59 (-1.8 ; 0.62)	0.47 (0.19 ; 1.17)
Diabetes mellitus	normoglycemic	0 (reference)	1 (reference)
	untreated diabetes	-2.29 (-4.71 ; 0.12)	0.41 (0.05 ; 3.11)
	insufficiently treated diabetes	-0.03 (-1.94 ; 1.88)	1.09 (0.35 ; 3.4)
	properly treated diabetes	**-3.03 (-5.46 ; -0.6)**	nc

* with corresponding [95%CIs]

nc: not computable
